# Effect of ultrasonic versus laser-activated irrigation on smear layer removal - An *in vitro* analysis

**DOI:** 10.6026/973206300223362

**Published:** 2026-06-30

**Authors:** Ojaswini Pawar, Ronak N Patel, Priyatam Maruti Karade, Chirantan Chowdhury, Rangoli Srivastava, Mohammed Mustafa

**Affiliations:** 1 Department of Conservative Dentistry and Endodontics, D.Y. Patil Dental School, Charholi Budruk, Lohegaon, India; 2 Department of Conservative Dentistry & Endodontics, Faculty of Dental Science, Dharmsinh Desai University, Nadiad, India; 3 Department of conservative dentistry and endodontics, Bharati Vidyapeeth (Deemed To Be University) Dental College and Hospital, Sangli, India; 4 Department of Conservative Dentistry and Endodontics, Sardar Patel post Graduate Institute of Dental and Medical Sciences, Lucknow, Uttar Pradesh, India; 5 Department of Public health dentistry, Faculty of dental sciences, SGT University, Haryana, India; 6 Department of Conservative Dental Sciences, College of Dentistry, Prince Sattam Bin Abdulaziz University, Al-Kharj 11942, Saudi Arabia

**Keywords:** Smear layer, passive ultrasonic irrigation, laser activated irrigation, erbium laser, root canal treatment

## Abstract

Elimination of the smear layer from root canal walls remains a clinical challenge, as residual debris can compromise sealer adaptation and endodontic treatment outcomes. Therefore, it is of interest to compare the efficacy of passive ultrasonic irrigation and erbium-doped yttrium aluminum garnet (Er:YAG) laser-activated irrigation in removing smear layer at different canal levels. Sixty extracted single-rooted human premolars were allocated to four groups: conventional syringe irrigation, passive ultrasonic irrigation and Er:YAG laser-activated irrigation at 1.0 W and 1.5 W using sodium hypochlorite and EDTA. Scanning electron microscopy evaluation demonstrated that both activation techniques significantly improved smear layer removal compared with conventional irrigation at all canal levels (p<0.001). Thus, Er:YAG laser-activated irrigation at 1.5 W showed superior smear layer removal in the apical third, suggesting its potential as an effective adjunct in contemporary endodontic practice.

## Background:

Effective endodontic treatment beliefs principally on complex chemo-mechanical advancement of the root canal complex including total removal of pulp tissue remains, microorganisms and unclean dentin of the complex three-dimensional canal structure [1]. The root canal wall instrumentation always produces an amorphous layer of organic and inorganic debris called the smear layer that sticks tenaciously to the surface of the canal and blocks the dentinal tubules orifices [2]. It is a layer that may be 1-5 micrometers in thickness, which is composed of fragmented odontoblastic processes, remnants of the pulp tissue, dentin fragments and microorganisms that gets compacted against the walls of the canals during rotary or reciprocating instrumentation [3]. The removal of smear layers has a controversial clinical implication in the endodontic literature. Those who support smear layer removal claim that the existence of the smear layer disrupts the close association of root canal sealers with dentin, which may lead to the development of microleakage channels that can invalidate the fluid-tight seal needed to achieve success in the long-term treatment [4]. Additionally, the smear layer could harbor viable bacteria in its structure, which could act as a reservoir of sustained infection, which may result in a treatment failure [5]. On the other hand, other researchers have proposed that a complete smear layer can decrease the permeability of dentin and inhibit the invasion of bacteria into dentinal tubules, but this view has been largely overtaken by the evidence that it is important to remove it [6]. In the modern endodontic practices, the removal of the smear layer is usually performed using a mixture of sodium hypochlorite and ethylenediaminetetraacetic acid. Sodium hypochlorite is known to have strong antimicrobial activity and dissolution of organic tissue constituents and the ethylenediaminetetraacetic acid binds calcium ions to dissolve the inorganic dentin constituent of the smear layer [ 7]. The efficacy of these irrigants however relies on the capability of interacting with canal surfaces especially in anatomically intricate areas such as fins, isthmuses and the apical third where the penetration of conventional syringe irrigation proves inadequate [8]. The natural constraints of passive syringe irrigation have led to the exploration of other agitation methods which are aimed at increasing the distribution of irrigants and cleaning efficiency. Passive ultrasonic irrigation is one of these developments which employs the effects of acoustic microstreaming and cavitation of free oscillating ultrasonic files to enhance the penetration of irrigant into canal distortion [9]. Many studies also have shown that passive ultrasonic irrigation has better cleaning effect than conventional delivery method using a syringe, especially in the apical third area, which is a challenging area to reach [10]. Another innovative alternative method is laser-activated irrigation, which is taking advantage of the photoacoustic and photothermal properties of laser energy, to produce strong agitation of the irrigant [11]. The erbium-doped yttrium aluminum garnet laser with its highest absorption in water at 2940 nanometers produces rapid expansion and collapse of vapor bubbles on activation in aqueous irrigating solutions [12]. The effect of this phenomenon is strong fluid flow and shear forces that have the ability to destabilize the debris and biofilm on the surfaces of the canals [14]. Recent studies have shown good outcomes of the photoacoustic streaming effect that is caused by the photon in the context of debris removal [14]. The comparative research studies using passive ultrasonic irrigation and laser-activated irrigation have also produced mixed results with some studies showing better performance of ultrasonic activation and others demonstrating better performance of laser-only methods [15]. Such deviations can be attributed to experiment technique, laser parameters, irrigation procedures and evaluation standards that are used by studies [16]. Furthermore, the impact of laser power parameters on the effectiveness of its cleaning and possible side effects should be evaluated in a systematic manner that will allow determining the most effective clinical parameters [17]. The apical third of the root canal system is especially challenging in the effective cleaning of the canal as the canal diameter is smaller, the canal system complex and there is the vapor lock phenomenon preventing the penetration of irrigants [18]. The removal of sufficient smear layer in this crucial area is required and apical seal is a major endodontic treatment outcome determinant [19]. The current literature is limited in comparing the efficacy of the methods of activation that are specifically focused on the regional variations in cleaning efficiency [20]. A knowledge gap still exists concerning the comparative efficacy of passive ultrasonic irrigation and erbium laser-activated irrigation at various power levels in the removal of the smear layer at various levels of the root canal [21] despite much research efforts that have been paid to the irrigation activation technologies. The current study was intended to determine the effectiveness of passive ultrasonic irrigation and erbium-doped yttrium aluminum garnet laser-activated irrigation at two power levels on the removal of smear layer on the wall of the root canal at the coronal, middle third and apical levels. The null hypothesis said that there would be no significant difference in the effectiveness of the smear layer removal of the methods of irrigation activation at any canal level. Therefore, it is of interest to evaluate and compare the effectiveness of passive ultrasonic irrigation and erbium-doped yttrium aluminum garnet laser-activated irrigation at different power settings on smear layer removal at the coronal, middle and apical thirds of the root canal system.

## Materials and Methods:

## Design of the study and ethical permission:

This *in vitro* experimental study was a control based experimental study carried out within the Department of Endodontics, Faculty of dentistry between February 2023 and November 2023.

## Sample size calculation:

The estimation of sample size was done with G*Power software version 3.1.9.7 and on the basis of effect sizes of past studies conducted on the activation techniques of irrigation. A minimum of 52 specimens was determined based on an assumed medium effect size (f=0.40), alpha error of 0.05 and statistical power of 0.85 and four experimental groups. A final sample of 60 teeth was used in order to allow the possibility of loss of a specimen during the preparation process and have sufficient statistical power. Selection and Preparation of Specimen. Sixteen teeth were used in the study of human single-rooted premolars in the oral surgery department after extraction due to orthodontic or periodontal causes. All patients were informed and gave their consent before the collection of teeth. Teeth were kept in 0.1% thymol solution at 4°C then used in experiments, all the work done within three months after extraction.

## Inclusion criteria:

[1] Single canal, single rooted premolar that is radiographically confirmed.

[2] Complete apices with no resorption.

[3] Straight and curved root canals below 10 degrees curvature as determined by Schneider.

[4] No history of prior endodontic therapy.

[5] Crown and root structure: No caries or fractures.

## Exclusion criteria:

[1] Calcified or obliterated canals of teeth.

[2] Internal or external root resorption.

[3] Roots shorter than 12 mm

[4] Cracks or structural defects.

Various canals or dramatic anatomical differences. A water-cooled diamond disc was used to decoronate selected teeth at the cementoenamel junction to standardize the length of the roots to 14 mm and to the apical foramen with a size 10 K-file (Dentsply Sirona, Ballaigues, Switzerland). Direct visualization was used in determining the working length with a size 15 K-file set at 1 mm below the apical foramine.

## Root canal instrumentation:

To facilitate standardization, all the specimens were instrumented by one expert operator.

The pre-preparation was done with ProTaper Gold rotary tools (Dentsply Sirona, Ballaigues, Switzerland) as the manufacturer suggests:

[1] SX file for coronal flaring

[2] S1 and S2 modification of files to working length.

[3] F1, F2 and F3 final apical preparation to working length ( 30/.09 taper)

Instrumentation was performed with 2 mL of 2.5% sodium hypochlorite between each change of the file, which was administered with a 30-gauge side-vented needle held 2 mm below the working length. Patency of canal was ensured during the instrumentation process with a size 10 Kfile. After instrumentation, 5 ml of 17% ethylenediaminetetraacetic acid was added to all the canals and left to liquefy the smear layer after 60 seconds, then 5 ml of distilled water was added to eliminate the rest of the chelating agent.

## Experimental groups:

Four experimental groups (n=15 each) were randomly assigned prepared specimens with the help of computer-generated randomization:

[1] Group 1 (Control) - Conventional Syringe Irrigation: Final irrigation procedure involved the following: 3 mL of 2.5% sodium hypochlorite after 60 seconds, 3 mL of 17% ethylenediaminetetraacetic acid after 60 seconds and 3 mL of 2.5% sodium hypochlorite after 60 seconds. The entire irrigants were administered by the 30-gauge side vented needle (NaviTip, Ultradent Products, South Jordan, UT, USA) 2 mm below the working length with slow up and down movement.

[2] Group 2 - Passive Ultrasonic Irrigation: Final irrigation was using the same irrigant order as Group 1. An ultrasonic unit (EMS Piezon Master 700, EMS, Nyon, Switzerland) with a size 15 stainless steel file ( Irrisafe, Satelec Acteon, Mérignac, France) was used to activate each irrigant application at power setting 3. The ultrasonic tip was aligned 1 mm below the working length and turned on three cycles of 20 seconds each time applying the irrigant, after which the syringe was refilled.

[3] Group 3 - Laser-Activated Irrigation (1.0 W): Last irrigation procedure used the same sequence of irrigant as Group 1. An Er:YAG laser (LightWalker AT, Fotona, Ljubljana, Slovenia) with a radial firing tip with 400 9mm was used to activate each irrigant application (PIPS tip, Fotona). Laser parameters: 1.0 W average power, 20 mJ pulse energy, 50 Hz repetition rate, 50 mu s pulse duration. The tip was placed in the pulp chamber without making any inlet to the canal and switched to three 20 s cycles with each application of the irrigant. A low-energy laser (1.5 W) was used to ablate the smoker and the control group.<|human|>Group 4 - Laser-Activated Irrigation (1.5 W):

Group 1 irrigation sequence was used as final irrigation protocol. Activation in Laser was done in a manner similar to that of Group 3 except using higher power levels: 1.5 W average power, 30 mJ pulse energy, 50 Hz repetition rate, 50 mcs pulse duration. Each application of irrigants was done in three 20-second activation cycles. Scanning Electron Microscopy The specimen preparation. After the irrigation procedures, 5 mL of distilled water was immediately applied to all the specimens followed by dry paper point. The buccal and lingual root surfaces were prepared with longitudinal grooves made using a diamond disc without going through the canal space. A chisel and mallet were then used to carefully cut the roots into half. Dehydration of the specimens was done using an ascending series of ethanol (50% ethanol, 70% ethanol, 80% ethanol, 90% ethanol, 95% ethanol and 100% ethanol), which was then followed by critical point drying. The dried samples were attached on aluminum stubs with the canal surface of the specimen facing upwards and sputter-coated with gold-palladium (20 nm thick). Evaluation was done using a field emission scanning electron microscope (JEOL JSM-7600F, JEOL Ltd., Tokyo, Japan) at standard magnifications.

## Scanning electron microscopy evaluation:

Three representative images were captured at each canal level (coronal, middle and apical thirds) at 2000x magnification for each specimen half. The coronal third was defined as 9-11 mm from apex, the middle third as 5-7 mm from apex and the apical third as 1-3 mm from apex. Images were obtained from the central area of each region, avoiding preparation artifacts and debris from the splitting procedure.

Two calibrated endodontists blinded to group allocation independently evaluated all images using the validated five-point smear layer scoring system:

[1] Score 1: No smear layer, all dentinal tubules open and visible

[2] Score 2: Minimal smear layer, majority of tubules open with small debris particles

[3] Score 3: Moderate smear layer, less than 50% of tubules visible, thin smear layer present

[4] Score 4: Heavy smear layer, few tubules visible, thick smear layer covering most surfaces

[5] Score 5: Complete smear layer, no tubules visible, uniform debris covering

Prior to experimental evaluation, inter-examiner calibration was performed using 30 reference images until kappa agreement exceeded 0.80. Discrepancies in scoring were resolved through consensus discussion.

## Statistical analysis:

Statistical analyses were performed using IBM SPSS Statistics version 28.0 (IBM Corporation, Armonk, NY, USA). Normality of data distribution was assessed using Shapiro-Wilk tests. As smear layer scores represented ordinal data with non-normal distribution, non-parametric statistical tests were employed. Kruskal-Wallis tests were used for overall group comparisons at each canal level, with post-hoc pairwise comparisons performed using Dunn's test with Bonferroni correction. Friedman tests were used to compare scores among canal levels within each group, with post-hoc Wilcoxon signed-rank tests. Inter-examiner reliability was assessed using weighted Cohen's kappa coefficient. Descriptive statistics were presented as mean ± standard deviation, median with interquartile range and frequency distributions. Statistical significance was established at p<0.05 for all analyses.

## Results:

All sixty specimens were successfully prepared and evaluated without loss during experimental procedures. Inter-examiner reliability demonstrated excellent agreement (weighted k=0.84, 95% CI: 0.78-0.90) and consensus scores were used for statistical analysis. Overall mean smear layer scores across all canal levels demonstrated significant differences among experimental groups (Table 1). The control group exhibited the highest mean score (3.71±0.82), indicating substantial residual smear layer, while laser-activated irrigation at 1.5 W achieved the lowest mean score (1.98±0.81), representing superior cleaning efficacy. Both activation techniques demonstrated significantly improved smear layer removal compared to conventional syringe irrigation (p<0.001). Comparison between activation techniques revealed that laser-activated irrigation at 1.5 W achieved significantly better overall smear layer removal compared to passive ultrasonic irrigation (p=0.018) and laser activation at 1.0 W (p=0.031). No significant difference was observed between passive ultrasonic irrigation and laser activation at 1.0 W (p=0.412). Analysis of smear layer scores by canal level revealed distinct patterns of cleaning efficacy among experimental groups (Table 2). In the coronal third, all three activation techniques achieved similar smear layer removal (mean scores: PUI 1.93±0.70, LAI 1.0 W 1.80±0.68, LAI 1.5 W 1.67±0.72), with no significant differences among activation methods (p=0.387). All activation techniques demonstrated significantly improved cleaning compared to control (mean score 3.27±0.88, p<0.001). In the middle third, activation techniques continued to show significant improvements over conventional irrigation. Laser-activated irrigation at 1.5 W achieved the lowest mean score (1.87±0.74), though differences among activation techniques did not reach statistical significance (p=0.089). The apical third demonstrated the greatest differentiation among irrigation techniques. Control specimens exhibited the highest mean smear layer scores (4.33±0.62), indicating substantial residual debris. Laser-activated irrigation at 1.5 W achieved significantly superior smear layer removal (mean score 2.40±0.83) compared to passive ultrasonic irrigation (mean score 3.07±0.80, p=0.012) and laser activation at 1.0 W (mean score 2.87±0.74, p=0.038) in this challenging region. Within-group analysis demonstrated that all groups showed significantly increased smear layer scores progressing from coronal to apical regions (Friedman test, p<0.001 for all groups), confirming the inherent difficulty of apical third cleaning regardless of irrigation method. Detailed analysis of score distributions provided additional insight into cleaning efficacy patterns (Table 3). The proportion of specimens achieving complete or near-complete smear layer removal (Scores 1-2) varied substantially among groups and canal levels. In the apical third, laser-activated irrigation at 1.5 W achieved Scores 1-2 in 60.0% of specimens, compared to 33.3% for laser activation at 1.0 W, 26.7% for passive ultrasonic irrigation and only 6.7% for conventional irrigation. The proportion of specimens with substantial residual smear layer (Scores 4-5) was 80.0% for control specimens in the apical third, compared to 40.0% for passive ultrasonic irrigation, 33.3% for laser activation at 1.0 W and 20.0% for laser activation at 1.5 W. Chi-square analysis confirmed significant associations between irrigation technique and score distribution at all canal levels (p<0.001), with the strongest differentiation observed in the apical third.

## Discussion:

The current study indicates that passive ultrasonic irrigation and erbium laser-activated irrigation are much more effective than the traditional syringe irrigation in smear layer removal and should be therefore rejected in favor of the alternative hypothesis. In addition, laser-enhanced irrigation with power of 1.5 W attained high cleaning effectiveness in the crucial apical third area and this implies that the technology may be of use in the modern endodontic procedures [22]. The fact that both methods of activations demonstrated a significant enhancement in smear layer removal is the validation of the inherent drawback of passive syringe irrigation with regard to sufficient canal debridement. Traditional needle irrigation has been based mostly on positive pressure and low exchange of irrigants that are not adequate to overcomes the vapor lock effect that entraps air in the apical canal area and denies sufficient contact of irrigant [23]. These limitations are conquered by the physical agitation delivered by ultrasonic and laser activation in different, but complementary, ways. Passive ultrasonic irrigation involves the creation of acoustic microstreaming by means of fast oscillation of ultrasonic file inside the irrigant solution. This micro streaming generates shear forces across the canal walls that dislodge adherent debris and increase penetration of irrigant in canal irregularity [24]. Past studies have also consistently shown that passive ultrasonic irrigation is more effective in removing smear layer than conventional irrigation with up to 30-50% improvement in the various experimental settings reported [25]. The laser-powered irrigation system is also fundamentally different, but it relies on the great absorption of water molecules to erbium laser light. As the laser pulse comes in contact with the aqueous irrigant, the superheating will happen rapidly giving vapor bubbles that grow and collapse in turn producing strong photoacoustic streaming effects [26]. The resulting phenomenon causes three-dimensional movement of irrigants in the canal system, which can extend to regions that cannot be activated by ultrasonic movement because of its dependence on actual mechanical vibration [27]. The enhanced performance of laser-activated irrigation of 1.5 W at the apical third can indicate the peculiarities of photoacoustic streaming in the narrow cavities. The bubble explosions produce large fluid displacement, less sensitive to tip positioning than ultrasonic activation, which could be the reason behind the increased apical cleaning [28]. Besides, the greater power state creates bigger and more energetic bubble events, which enhance the forces of shear on adherent debris [29]. The clinical implications of the power-dependent laser-activated irrigation are significant during 1.0 W to 1.5 W of power. Although increased power levels enhanced cleaning efficacy, the choice of laser parameters needs to be effective and reduce the risk of some undesirable effects such as thermal damage of periradicular tissues and excessive extrusion of the irrigant [30]. The parameters used in this study were not exceeded by accepted safety standards of the use of endodontic lasers, but the clinical use should be based on the specifics of each case. The progressive reduction of cleaning efficacy between coronal and apical regions in all groups can be attributed to the existing knowledge about the irrigation difficulties in the apical third. These factors include the smaller diameter of the canal and the density of the dentin and the complexity of the apical region anatomy, which inherently hinders the removal of the debris despite the technique of irrigation [31]. Nevertheless, the size of this regional disparity was significantly decreased with laser-activated irrigation at 1.5 W, indicating that the size of laser parameters can, in part, be used to circumvent these anatomical constraints. The fact that all the methods of activation do produce a comparable result in the coronal and middle thirds implies that more than one method can be used to produce sufficient cleaning in these more accessible areas. This observation implies that the main distinguishing variable in irrigation methods is the apical third, which must be used in clinical decision-making in case the apical seal integrity is of great concern to the particular case [32]. The results of this study are consistent with the recent meta-analyses that have found laser-activated irrigation to have comparable and even more effective cleaning efficiency than ultrasonic activation in some parameters [33]. Nonetheless, it is not possible to directly compare it with other studies because of methodological differences such as various laser systems, power parameters, irrigant protocols and evaluation criteria used in various studies [34]. There are a number of methodological issues that should be mentioned when these findings are interpreted. Although the *in vitro* experimental design can offer standardized and reproducible conditions that are needed in controlled comparison, it might not necessarily recreate clinical conditions such as presence of vital tissues, inflammatory processes and the complex biofilm communities found in infected root canals [35]. Although the single-rooted premolar model is suitable to be used in terms of standardization, it might not be associated with the performance of anatomically complex molars, which have multiple canals and isthuses. The scanning electron microscopy analysis, which is generally used in assessing the scanning of the smear layer, only gives information on the surface features on a particular location but does not give the complete three dimensional picture of the cleaning patterns within the canal system [36]. Also, ordinal scoring, though valid and reliable, might fail to reflect minor variations in cleaning efficacy that might affect clinical outcomes. Clinical validation studies that measure the effects of irrigation activation methods on the outcome of treatment in real patient groups should also be conducted in the future. A study that investigates the antimicrobial effect of various activation modalities and their ability to penetrate organised biofilm structures would be a significant supplementary information [37]. Also, the research of combined methods of activation and optimization of laser parameters in a particular clinical scenario can reveal further ways of enhancing the effectiveness of irrigation [39]. Activated irrigation techniques demonstrate improved effectiveness in removing microorganisms, dentinal debris and smear layer from the root canal system compared with conventional irrigation methods [39].

## Conclusion:

Irrigant activation techniques significantly enhance smear layer removal compared with conventional syringe irrigation in root canal therapy. Improved cleaning is particularly evident in the apical region, which is critical for treatment success. Thus, the use of advanced activation methods may therefore contribute to more predictable endodontic outcomes when applied judiciously.

## Figures and Tables

**Table 1:** Overall smear layer scores by experimental group

**Group**	**Mean ±SD**	**Median (IQR)**	**Score Distribution n (%)**	**p-value**
			Score 1-2	Score 3
Control (Syringe)	3.71 ±0.82	4.0 (3.0-4.0)	8 (17.8)	14 (31.1)
PUI	2.42 ±0.79	2.0 (2.0-3.0)	26 (57.8)	13 (28.9)
LAI 1.0 W	2.31 ±0.76	2.0 (2.0-3.0)	28 (62.2)	12 (26.7)
LAI 1.5 W	1.98 ±0.81	2.0 (1.0-2.0)	35 (77.8)	7 (15.6)
*Kruskal-Wallis test with Dunn's
post-hoc comparison a: Significant difference
compared to Control;
b: Significant difference compared to
PUI: Passive ultrasonic irrigation;
LAI: Laser-activated irrigation;
SD: Standard deviation;
IQR: Interquartile range n=45 observations
per group (15 specimens x 3 canal levels)

**Table 2:** Smear layer scores by canal level and experimental group

**Canal Level**	**Control**	**PUI**	**LAI 1.0 W**	**LAI 1.5 W**	p-value*
	Mean ±SD	Mean ±SD	Mean ±SD	Mean ±SD	
Coronal Third	3.27 ±0.88a	1.93 ±0.70b	1.80 ±0.68b	1.67 ±0.72b	<0.001
Middle Third	3.53 ±0.74a	2.27 ±0.70b	2.27 ±0.80b	1.87 ±0.74b	<0.001
Apical Third	4.33 ±0.62a	3.07 ±0.80b	2.87 ±0.74bc	2.40 ±0.83c	<0.001
p-value†	<0.001	<0.001	<0.001	0.002	
*Kruskal-Wallis test for
between-group comparison at each
level †Friedman test for within-group
comparison across levels Different superscript
letters indicate significant differences
(p<0.05) in post-hoc analysis
PUI: Passive ultrasonic irrigation;
LAI: Laser-activated irrigation;
SD: Standard deviation n=15 specimens
per group at each canal level

**Table 3:** Score distribution by canal level and experimental group

**CanalLevel/Score**	**Controln(%)**	**PUIn(%)**	**LAI1.0Wn(%)**	**LAI1.5Wn(%)**	p-value*
CoronalThird					
Score1-2	4(26.7)	11(73.3)	12(80.0)	13(86.7)	<0.001
Score3	5(33.3)	3(20.0)	2(13.3)	1(6.7)	
Score4-5	6(40.0)	1(6.7)	1(6.7)	1(6.7)	
MiddleThird					
Score1-2	3(20.0)	10(66.7)	10(66.7)	13(86.7)	<0.001
Score3	5(33.3)	4(26.7)	4(26.7)	1(6.7)	
Score4-5	7(46.7)	1(6.7)	1(6.7)	1(6.7)	
ApicalThird					
Score1-2	1(6.7)	4(26.7)	5(33.3)	9(60.0)	<0.001
Score3	2(13.3)	5(33.3)	5(33.3)	3(20.0)	
Score4-5	12(80.0)	6(40.0)	5(33.3)	3(20.0)	
*Chi-square test
PUI: Passive ultrasonic irrigation;
LAI: Laser-activated irrigation
n=15 specimens per group at each canal level
